# Impact of depression on the perception of fatigue and information processing speed in a cohort of multiple sclerosis patients

**DOI:** 10.1186/s40359-023-01235-x

**Published:** 2023-07-14

**Authors:** Madia M. Biasi, Alessia Manni, Ilaria Pepe, Chiara Abbatantuono, Daphne Gasparre, Pietro Iaffaldano, Marta Simone, Maria F. De Caro, Maria Trojano, Paolo Taurisano, Damiano Paolicelli

**Affiliations:** 1 Department of Translational Biomedicine and Neuroscience, University of Bari “Aldo Moro”, Piazza G. Cesare, 11, Bari, 70121 Italy; 2 Department of Biomedical Sciences and Human Oncology, University of Bari “Aldo Moro”, Piazza G. Cesare, 11, Bari, 70121 Italy

**Keywords:** Multiple sclerosis, Cognition, Information processing speed, depression, fatigue

## Abstract

**Background:**

Information processing speed is commonly impaired in people with multiple sclerosis (PwMS). However, depression and fatigue can affect the cognitive profile of patients: fatigue has a negative impact from the disease’s earliest stage and a reduced information processing speed is often associated with higher levels of depression. Therefore, the aim of this study was to investigate the correlations between information processing speed and physical fatigue in a cohort of Italian PwMS from a single center, considering the effect of depression.

**Methods:**

Two hundred (W = 128; mean age = 39.83 years; SD = 11.86) PwMS, from the Bari University Hospital, underwent testing for processing speed (Symbol Digit Modalities Test [SDMT]), fatigue level (Fatigue Severity Scale [FSS]), and depression (Beck’s Depression Inventory [BDI]).

**Results:**

Statistically significant correlations emerged between SDMT and FSS, SDMT and BDI, FSS and BDI. Mediation analyses revealed that while physical fatigue had no significant direct negative effect on information processing speed (z=-0.891; p > 0.05), depression predicted the relationship between fatigue and information processing speed (z=-2.181; p < 0.05).

**Conclusion:**

Our findings showed that cognitive performance at SDMT was not affected by patients’ perceived level of physical fatigue, but by depression. The presence of a high BDI score mediates the physical fatigue on cognitive performance impact.

## Background

Multiple Sclerosis (MS) is a chronic, inflammatory, demyelinating, and neurodegenerative disease of the central nervous system characterized by demyelination and axonal damage [1] and it is clinically characterized by relapses, remissions, and progression of disability over time [2]. Cognitive impairment (CI) affects 40–60% of PwMS, although it is less severe than in those with other neurodegenerative disorders [3]. The cognitive domains most affected in MS are complex attention, memory and learning, and executive functions [2]. The slowdown in the information processing speed seems to be the most frequent cognitive alteration in MS and represents a clear cognitive deficit, which could contribute to the presence of impairments in other cognitive domains [4] affecting the performance of PwMS in probing executive functions tasks, thus explaining the deficits they achieve in this domain [3, 5]. When assessing and defining the cognitive functioning of PwMS, it is also necessary to consider some confounding factors, such as depression, anxiety, fatigue, and sleep disturbances, because they could affect the cognitive profile [2]: increased depressive symptoms, for example, in PwMS over time have been associated with decreased processing speed [6]. The incidence of depressive symptoms in PwMS is believed to be 50%, and it is greater in females and individuals under the age of 45 [6]; the aetiology of depression includes immune-inflammatory, immune-genetic, psychological, and specific MS brain damage as well as other elements [7]. Slower information processing speed was associated with higher levels of depression [8], fatigue [9], lower verbal fluency [10], fewer words and digits remembered [11], and poorer visual-spatial information recall [10]. The association between processing speed and more difficult tasks, such as immediate word recall and word list learning, was mostly influenced by depression and physical fatigue [12]. Additionally, a strong negative correlation has been proved between fatigue and processing speed in MS [13]. MS fatigue is defined as a subjective lack of physical and/or mental energy that is perceived by the subject and caregiver as interfering with daily activities [14].

Based on the reviewed literature, the primary aim of our work was to examine information processing speed in a cohort of PwMS and to assess if the perceived fatigue is directly correlated with information processing speed. Moreover, we investigated if depressive symptoms may interfere with the effects of physical fatigue at the Symbol Digit Modalities Test (SDMT).

## Methods

### Study sample and procedures

This cross-sectional study included subjects with a new diagnosis of MS accessing the Center for the Diagnosis and Treatment of Translational Biomedicine and Neurosciences of the Bari Polyclinic from March and October 2021. Inclusion criteria were: (1) MS diagnosis according to McDonald’s revised criteria of 2017; (2) age at the enrolment between 16 and 70 years; (3) being naïve to any Disease Modifying Drugs for MS. The following were considered exclusion criteria: (1) intake of steroid therapies (for clinical and/or radiological relapse of the disease) in the 30 days prior to access in our unit; (2) use of antidepressants, benzodiazepines, and neuroleptics; (3) chronic drug or alcohol intake. Patients were evaluated by psychologists, specially trained in neuropsychology. The assessment was conducted in a single session lasting approximately 10 min with a standardized methodology, before receiving diagnosis. The setting was face-to-face between examiner and examinee in special testing rooms. After a preliminary interview, multilevel tests and a domain-specific assessment investigated were administered. The study protocol was approved by the institutional ethics committee and all participants provided written informed consent prior to their participation.

### Measures

Demographic, clinical, and anthropometric data were collected for all subjects. The following medical diagnostic examinations and neuropsychological evaluation procedures were performed:

### Neuropsychological evaluation

Cognitive performance, in particular information processing speed, was assessed through the Symbol Digit Modalities Test (SDMT), included in the Brief Repeatable Battery of Neuropsychological Test (RBR-n) [15]. The Symbol Digit Modified Test is a cognitive processing speed test traditionally administered by neuropsychologists for both screening and assessment purposes. Patients are provided with 9 symbol-digit pairs, followed by several lines of symbols without the corresponding number. The patient is required to match each symbol to the corresponding number, during a total timespan of 90s.

### Assessment of fatigue and depression

Physical fatigue was assessed through Fatigue Severity Scale (FSS) [16]. It is a 9-item scale that measures the severity of perceived fatigue and its impact on activities of daily living, considering the last week. The items are scored on a 7-point scale ranging from 1 = strongly disagree to 7 = strongly agree. A score greater than 4.5 denotes the presence of physical fatigue.

The assessment was completed with the administration of a self-completed scale to assess depression (Beck’s Depression Inventory - BDI) [17], a multiple-choice self-report inventory consisting of 21 questions that investigates the presence and severity of possible depressive symptomatology. The subject is asked to refer to the 4 weeks prior to administration to describe his or her mood, marking a cross on the most representative option among the 5 proposed. The instrument considers numerous factors, such as: overall mood; pessimism; sense of failure; lack of satisfaction; guilt; sense of punishment; self-hatred; self-accusations; desires for self-punishment; crying; irritability; lack of interest; indecision; body image; work inhibition; sleep disturbance; fatigability; loss of appetite; weight loss; somatic concerns; loss of libido.

### Statistical analysis

Descriptive statistics were used to describe participants’ demographic and clinical data and their levels of depression, fatigue, and cognitive ability.

For the entire cohort, characteristics were analyzed in terms of mean ± standard deviation (SD) and percentile and categorical variables have been presented as frequencies (proportions).

The distribution of scores of the SDMT, BDI, and FSS were analyzed using the Kolmogorov-Smirnov test. In our sample, for all tests, the data did not correspond to a normal distribution. Therefore, Mann Whitney’s nonparametric U- test was used to compare measures with respect to gender and age and the significance level was set at 5% (two-tailed test).

Internal correlation analysis between the fatigue index, depression, and information processing speed was performed using the Bravais-Pearson linear mode coefficient to examine the interdependence between cognitive, perceived fatigue, and psychological symptoms. In a secondary analysis, these relationships were investigated while controlling for age. To this end, partial correlations (two-tailed, a = 0.05) were performed between the above variables, controlling for participants’ ages. As a precaution against type I error inflation, the procedure of Benjamini-Hochsberg (Benjamini & Hochberg, 1995) to control the False Discovery Rate (FDR). To investigate the potential indirect effect of depression on the association between physical fatigue and response in information processing, a mediation analysis was used. Specifically, we considered participants’ physical fatigue as the independent variable, SDMT score as the dependent variable, and BDI scores as the mediating variables. Direct and indirect effects were estimated for participants with an overt diagnosis of multiple sclerosis.

The level of statistical significance was set at p < 0.05. All statistical analyses were performed with the statistical software Jasp 0.16.3 (JASP Team, 2021).

## Results

### Sample characteristics

A total of 200 PwMS were included in the study. Participants were mostly women (n = 128), with a mean age of 39.83 years (SD = 11.86). In total, 69.4% were diagnosed as RRMS. The mean clinical onset of MS was about 10 years (SD = 9.90) (Table 1), while patients received the diagnosis only after neuropsychological evaluation.

**Table 1:** Demographic and clinical characteristics of Multiple Sclerosis (MS) patients

	N (%)	mean	SD	Range
Age		40,38	12,40	18–70
Gender				
Male (*n =* 72*)*		41.35	13.32	18–70
Female (*n* = 128)		39.83	11.86	18–70
Relapsing-Remitting Multiple Sclerosis (RR-MS)	127 (69.4)			
MS clinical onset (years)		10.21	9.90	1–33
Number of lesions	162	5.74	3.67	0–15

Across all participants, the mean score of SDMT was 47,79 (SD = 13.17), clinically significant fatigue was reported by 28.6%, and 44.4% showed a BDI score ≥ 10, significant for depression.

As expected, patients older than 35 years old such as women performed worse than younger and male subjects on SDMT (U = 2.589; p < 0.001; U = 6.096) (Table 2).

**Table 2:** Mean values and percentages of psychological and cognitive variables in pwMS

	Mean	N (%)	Average rank	U*	p
**FSS**	3.30 ± 1.75				
Gender				4436.5	0.73
*M*		72	101.07		
*W*		127	98.12		
Age intervals				4.957	0.157
*18–35*		67	92.01		
*≥ 36*		132	104.06		
**SDMT**	47.80 ± 13.18				
Gender				6096.5	0.000**
*M*		72	121.17		
*W*		125	86.23		
Age intervals				2.689.5	0.000**
*18–35*		66	123.76		
*≥ 36*		131	86.53		
**BDI**	9.75 ± 7.89				
Gender				3996.5	0.185
*M*		71	92.29		
*W*		127	103.53		
Age intervals				4.145.5	0.473
*18–35*		68	103.54		
*≥ 36*		130	97.39		
**FSS**					
*Absence*		142 (71.4)			
*Presence*		57 (28.6)			
**BDI**					
*Absence*		110 (55.6)			
*Presence*		88 (44.4)			

**Notes**: *Mann-Whitney U; ** p < 0.01. Mean values and percentages of psychological and cognitive variables and comparison of Physical Fatigue (Fatigue Severity Scale, FSS), Depression (Beck’s Depression Inventory, BDI), and Information Processing Speed (Symbol Digit Modalities Test, SDMT) between women and men and between low- and high-aged people in the MS group

### Correlation and meditation analysis

Correlations between information processing speed, depressive symptoms and fatigue are shown in Table 3. Greater physical fatigue was associated with worse performance in information processing speed (r=-0.181; p < 0.001) and higher levels of depression (r = 0.562; p < 0.001). Similarly, greater depressive symptomatology was associated with attentional-executive deficits (r=-0.229; p < 0.001) (Table 3).

**Table 3:** Correlation coefficients between fatigue, depression and information processing speed and age in MS group

	FSS	AGE	SDMT	BDI
**FSS**				
Pearson correlation	1	0.127	− 0.181	− 0.562
Sig. (2-tailed) with Benjamini-Hocberg correction		0.074	0.11	0.000**
N	200	97	97	97
**AGE**				
Pearson correlation	0.127	1	− 0.381	0.010
Sig. (2-tailed) with Benjamini-Hocberg correction	0.074		0.000**	0.890
N	97	200	97	97
**SDMT**				
Pearson correlation	− 0.181	− 0.381	1	− 0.229
Sig. (2-tailed) with Benjamini-Hocberg correction	0.000**	0.011		0.001
N	97	97	200	97
**BDI**				
Pearson correlation	− 0.562	0.010	− 0.229	1
Sig. (2-tailed) with Benjamini-Hocberg correction	0.000**	0.890	0.001	
N	97	97	97	200

**Notes**: ** p < 0.01

To confirm that the associations between mood/fatigue and participants’ cognitive functioning were not explained by age, we performed partial correlations to eliminate the potential influence of age (Table 4).

**Table 4:** Partial correlation between FSS, BDI and SDMT by “controlling” the age’s effect in MS group

AGE	FSS	SDMT	BDI
**FSS**			
Pearson correlation	1	− 0.142	0.567
Sig. (2-tailed) with Benjamini-Hocberg correction		0.49	0.000**
N	200	97	97
**SDMT**			
Pearson correlation	− 0.142	1	− 0.241
Sig. (2-tailed) with Benjamini-Hocberg correction	0.49		0.001
N	97	200	97
**BDI**			
Pearson correlation	0.567	− 0.241	1
Sig. (2-tailed) with Benjamini-Hocberg correction	0.000**	0.001	
N	97	97	200

**Notes**: ** p < 0.01

Notably, the direction and magnitude of the associations remained largely unchanged when the variance associated with age was divided.

In addition, partial correlations between SDMT score, depressive symptomatology, and physical fatigue were significant: SDMT versus BDI (r=-0.241; p < 0.001); versus FSS (r=-0.142; p < 0.05); BDI versus FSS (r = 0.567; p < 0.001).

A mediation model with BDI score (M), SDMT score (Y), and physical fatigue (X) explained 5.7% of the variance (R2) of SDMT score and 31.1% of depression. As expected, a decrease in SDMT score was significantly associated with an increase in fatigue and depressive symptomatology (Sobel’s test revealed that the indicated reduction was significant) (Table 3). The total and direct effects of fatigue on SDMT scores were − 0.103 and − 0.043, respectively, of which only the former was statistically significant, i.e., each additional score at FSS, in addition to the increase in depressive symptomatology, contributed to the total decrease of 0.103 units in SDMT score but not directly (z=-0.891; p > 0.05). In contrast, the indirect effect (FSS → Depression → SDMT) was significant (z=-2.181; p < 0.05), with an indirect/total negative effect of -0.06. These results suggest that depression totally mediates the relationship between physical fatigue and information processing speed: as physical fatigue increases, depression increases, and as depression increases, information processing speed decreases (Table 5).

**Table 5:** Mediation analyses results

					Estimate	95% CI Lower	95% CI Upper	z-value	p**
**Direct effects**									
FSS	→	SDMT			-0.043	-0.137	0.051	-0.891	0.373
**Indirect effects**									
FSS	→	BDI	→	SDMT	-0.060	-0.115	-0.006	-2.181	0.029
**Total effects**									
FSS	→	SDMT			-0.103	-0.182	-0.025	-2.576	0.010
**Path coefficients**									
BDI	→	SDMT			-0.188	-0.352	-0.024	-2.242	0.025
FSS	→	SDMT			-0.043	-0.137	0.051	-0.891	0.373
FSS	→	BDI			0.322	0.256	0.388	9.558	0,000**

**Notes**: ** p < 0.01Direct effects report the relationship between fatigue and the symbol digit modality test not considering the depression index. Indirect effects report the mediation role of depression. Total effects report both the direct and mediated effect

## Discussion

The prevalence of cognitive impairment in MS is estimated between 54% and 65% in clinic-based studies [18] with information processing speed, sustained attention, memory, and visuospatial perception as most frequently impaired domains. These may impact significantly and independently on employment and social functioning [19], therefore there is a strict need to test patients for cognitive impairment from the early stages of the disease. Several batteries have been developed to detect cognitive impairment in MS, however, most of them require a lot of time (0.5–2 h) to be administered. The BICAMS recommends the use of the Symbol Digit Modalities Test (SDMT) when only 5 min are available [20] which has also been reported as the most sensitive test when comparing healthy controls and PwMS and for cognitive monitoring in patients only [21, 22]. However, few studies have directly evaluated the impact that Fatigue and depressive symptoms can have on SDMT.

We sought to explore the relationship between physical fatigue, depression, and information processing speed in PwMS, hypothesizing that depression is predictive of the relationship between physical fatigue and cognitive function. Our main objective was to explore the role of depression as a mediator of the relationship between FSS and SDMT considered the most sensitive individual cognitive measure for the disease [23] and a measure of information processing speed [24].

Overall, our results indicate a consistent relationship in which increased physical fatigue was associated with poorer processing speed performance. In fact, fatigue in MS is generally considered a very common invisible symptom [25], so much so that the National MS Society reports it in about 80% of patients [14]. Fatigue is characterized by a loss of energy that can limit mobility, participation in social life, and physical function [26], as well as having a psychosocial impact. It has been proved that the information processing speed were worse in PwMS with a higher psychological dimension of fatigue [9]. In addition, people with fatigue experience this symptom with subjective and unique cognitive and physical components [27].

Although distinct from depression, fatigue symptoms overlap and are highly correlated even when somatic depressive symptoms are omitted [28, 29]. Depression can predict later fatigue and fatigue can predict later depression [29].

Similarly, regarding depression, our results showed that increased depressive symptoms were associated with worse performance in SDMT and increased physical fatigue [9, 30, 31]. PwMS commonly experience depression that affects their cognitive and physical functions [32, 33].

Based on these direct correlations, for the first time to our knowledge, in our study a mediation analysis was carried out considering depression as a potential mediator in the model, in order to understand whether it was depressive symptoms that influenced the relationship between cognitive performance and physical fatigue. Mediation analyses revealed that physical fatigue had no direct negative effect on information processing speed. In contrast, physical fatigue predicted depression, which in turn predicted decreased information processing speed.

Existing literature suggests that depression affects more than 29% of PwMS, resulting in an impact on their quality of life, altered use of health care services, increased hospitalizations, and mortality. Depression is also correlated with impairment in the domains of attention and processing speed: specifically, patients complain of cognitive changes accompanying mood symptoms, apparently reinforced by feedback to objective tests. Moreover, there is some agreement between the clinical presentation of depression, characterized by anhedonia, lack of energy, decreased motivation, and reduced motor activity [34], and the associated cognitive profile, namely decreased attention and slowed information processing speed [35]. The “cognitive effort hypothesis” theory in the psychiatric literature had previously attempted to investigate the observed relationships between depression and cognition, proposing the conclusion that depression was specific to performance in tasks requiring effort in information processing, rather than those requiring automatic processing [35–37]. In contrast, as shown in our study, physical fatigue, in the existing literature, has no impact or influence on cognitive performance of PwMS, attributing the latter to both the neurodegenerative nature of the disease [38, 39] and depressive symptoms [30]. In fact, some studies show that it is the combination of fatigue and mood that accounts for a substantial portion of the total variance in cognitive deficits [38, 40].

### Limitations

Some limitations should be kept in mind when interpreting our findings. The cross-sectional design, based on self-report scores of fatigue and neuropsychiatric symptoms, was conducted in a single center with a relatively small number of patients (albeit well representative for age, sex, and EDSS of the quite typical MS population). In addition, only the relationship between depression, physical fatigue and information processing speed was investigated: it would be interesting to see if these results are also confirmed by examining cognitive fatigue. Based on our preliminary findings, and on the existing data, future studies should aim to comprehensively assess the cognitive profile of PwMS to investigate both the most impaired domains in the disease and the possible impact of anxiety-depressive symptoms and perceived physical fatigue on cognitive performance. Interestingly would be to observe parallel changes in information processing speed, physical fatigue, and depression measures in larger cohorts and during long-term follow-up, which may be a future objective of this cross-sectional preliminary work.

## Conclusions

In our experience there is a link between FSS and BDI, SDMT and FSS, BDI and SDMT. However, as proved by our mediation analysis, the correlation between FSS and SDMT only becomes statistically significant with the mediation of BDI. As a result, performance on the information processing speed test is affected by the effect of depressive symptoms that cause a feeling of physical fatigue. Overall, despite the preliminary nature of the findings obtained from a relatively small sample, the mediation analyses conducted in our study shed light on the interplay between depression, fatigue, and information processing, allowing for the estimation of direct and indirect effects of fatigue on attentional performance and providing insights into the psychological mechanisms underlying the association between the explanatory variable and cognitive outcomes in PwMS, while accounting for the complexity of MS clinical manifestations. Therefore, a multi-dimensional and multi-focusing approach should be warranted to PwMS to improve these, often hidden symptoms: if mood disorders are not appropriately treated, they may cause a lack of autonomy and poorer cognitive performances, resulting in a decreased quality of life in PwMS.

## Data Availability

The datasets used and/or analysed during the current study are available from the corresponding author on reasonable request.

## List of Abbreviations

SDMT: Symbol Digit Modalities Test
FSS: Fatigue Severity Scale
BDI: Beck’s Depression Inventory
